# Montreal cognitive assessment reflects cognitive reserve

**DOI:** 10.1186/s12877-018-0951-8

**Published:** 2018-10-30

**Authors:** Jae Myeong Kang, Young-Sung Cho, Soowon Park, Byung Ho Lee, Bo Kyung Sohn, Chi Hyun Choi, Jeong-Seok Choi, Hee Yeon Jeong, Seong-Jin Cho, Jae-Hong Lee, Jun-Young Lee

**Affiliations:** 1 Department of Psychiatry, Gil Medical Center, Gachon University College of Medicine, Incheon, Republic of Korea; 2 Department of Psychiatry, SMG-SNU Boramae Medical Center, Boramae-Ro 5-Gil, Shindaebang-dong, Dongjak-gu, Seoul, Republic of Korea; 3 Department of Psychiatry and Behavioral Science, Seoul National University College of Medicine, Boramae-Ro 5-Gil, Shindaebang-dong, Dongjak-gu, Seoul, Republic of Korea; 4 Department of Education, Sejong University, Seoul, Republic of Korea; 5 Department of Psychology, Salisbury University, Salisbury, Maryland USA; 6 Department of Neurology, Asan Medical Center, University of Ulsan College of Medicine, Seoul, Republic of Korea

**Keywords:** MoCA, Cognition, Cognitive reserve, Dementia, Mild cognitive impairment

## Abstract

**Background:**

The Montreal Cognitive Assessment (MoCA) is known to have discriminative power for patients with Mild Cognitive Impairment (MCI). Recently Cognitive Reserve (CR) has been introduced as a factor that compensates cognitive decline. We aimed to assess whether the MoCA reflects CR. Furthermore, we assessed whether there were any differences in the efficacy between the MoCA and the Mini-Mental State Examination (MMSE) in reflecting CR.

**Methods:**

MoCA, MMSE, and the Cognitive Reserve Index questionnaire (CRIq) were administered to 221 healthy participants. Normative data and associated factors of the MoCA were identified. Correlation and regression analyses of the MoCA, MMSE and CRIq scores were performed, and the MoCA score was compared with the MMSE score to evaluate the degree to which the MoCA reflected CR.

**Results:**

The MoCA reflected total CRIq score (CRI; *B* = 0.076, *P* < 0.001), CRI-Education (*B* = 0.066, *P* <  0.001), and CRI-Working activity (*B* = 0.025, *P* = 0.042), while MMSE reflected total CRI (*B* = 0.044, *P* <  0.001) and CRI-Education (*B* = 0.049, *P* <  0.001) only. The MoCA differed from the MMSE in the reflection of total CRI (*Z* = 2.30).

**Conclusion:**

In this study, we show that the MoCA score reflects CR more sensitively than the MMSE score. Therefore, we suggest that MoCA can be used to assess CR and early cognitive decline.

**Electronic supplementary material:**

The online version of this article (10.1186/s12877-018-0951-8) contains supplementary material, which is available to authorized users.

## Background

In recent years, the number of patients with dementia has increased worldwide. This increase emphasizes the importance of early detection and treatment of dementia. Therefore, the development and standardization of effective screening tools are required. The Montreal Cognitive Assessment (MoCA) is known to distinguish patients with Mild Cognitive Impairment (MCI) from the normal population [1]. MoCA has shown higher sensitivity in detecting cognitive decline than the Mini-Mental State Examination (MMSE) [2], another common clinical screening tool for Alzheimer’s disease (AD). Previous studies have indicated that the MoCA exhibits high sensitivity and specificity in other languages as well. Moreover, MoCA is not only highly sensitive in identifying patients with AD, but also non-AD patients who demonstrate behavioral variants of frontotemporal dementia [3], dementia associated with Parkinson’s disease [4], and vascular dementia [5].

Cognitive Reserve (CR) is a concept based on the plasticity of the brain. CR is believed to counter the effects of aging or brain damage. It has been suggested that environmental factors play an important role in the onset of AD. Moreover, a meta-analysis reported that higher CR lowers the risk for incidence of dementia to 54% [6]. CR is associated with diverse factors of life experience such as higher intellectual quotient (IQ), education, occupational complexity and duration, and lifestyle [7]. Several measures have been developed to assess CR using these variables. The Cognitive Reserve Index questionnaire (CRIq), which has been developed by Nucci et al. [8], has advantages over other measures assessing comprehensive CR; CRIq measures 3 subdomains, i.e., education, occupation, and leisure activities, which are the most used proxies of CR [9]. Unlike other measurements, which only evaluate current activities of 1 or 2 domains, CRIq considers activities from all 3 subdomains throughout adulthood, including the frequency of the activities.

As the MoCA exhibits higher sensitivity than the MMSE in cognitive decline in the early stages of AD, the MoCA might be more sensitive to factors such as age, sex, and CR than MMSE. Recently, a study reported that educational domain in CR can affect MoCA and MMSE scores in patients with MCI [10]. However, to the best of our knowledge, no prior studies have assessed the degree to which MoCA reflects CR. Moreover, comparisons of the level at which MoCA and MMSE reflect CR are lacking. In the present study, we examined the associations of demographic factors (sex, age, and education) and CR with MoCA and compared the level of reflection of CR in MoCA and MMSE using CRIq as a comprehensive measure of CR.

## Methods

### Subjects

Subjects were recruited from a community-based center from March 2013 to June 2016 through recruitment announcements. Assessments of their physical and neuropsychiatric disorders were performed by 2 community dementia center consultant psychiatrists with 6 years and 15 years of experience, respectively.

Subjects with dementia or any mental or physical disease that may affect cognitive functioning, such as alcohol or other substance abuse, history of infarction, any evidence of central nervous system disorders or brain damage were excluded. Patients with severe major depressive disorders, altered state of consciousness like delirium, severe loss of hearing or sight, or language disorders were also excluded. However, patients with general medical problems, well-controlled diabetes, essential hypertension, or mild impairment of vision or hearing due to aging were not excluded if the impairment did not restrict their ability to perform the tests. Exclusion criteria were determined by psychiatrists on the basis of diagnostic criteria in the Diagnostic and Statistical Manual of Mental Disorders, fourth Edition (DSM-IV) [11].

A total of 221 subjects participated in this study and the age of subjects ranged from 60 to 90 years. Informed consent was obtained from all participants and the study was approved by the institutional review board of SMG-SNU Boramae Medical Center.

### Data collection

Clinical neuropsychological tests (MoCA and MMSE) were administered to all subjects by mental health center specialists and professional dementia researchers (nurses, clinical psychologists) who had experience with the tests for an average of 10 years. CR was assessed using the self-reported CRIq completed by the subjects and interviews with the subjects’ close family members.

### Measurements

#### Korean version of Montreal cognitive assessment (MoCA-K)

MoCA is a screening instrument to detect MCI developed by Nasreddine et al. Administration of MoCA takes about 10 to 15 min. Higher scores indicate better cognition; the maximum score is 30 [2]. There are 12 items for cognitive domains; memory is tested by a short-term memory recall task (5 points); visuospatial ability is tested using a clock-drawing test (CDT; 3 points) and a 3-dimensional cube copy (1 point); executive function is tested using a trail-making test, part B (TMT-B; 1 point), a phonemic fluency task (1 point), and a 2-item verbal abstraction task (2 points); attention, concentration, and working memory is tested using a sustained attention task (1 point), a serial subtraction task (3 points), and digits forward and backward tasks (1 point each); language is tested using a 3-item confrontation naming task with low-familiarity animals (lion, camel, rhinoceros; 3 points) and repetition of 2 syntactically complex sentences (2 points); orientation in time and place was also tested (6 points). MoCA-K was standardized for Koreans. It should be taken into consideration that the words for the short-term memory recall task and the TMT-B were replaced by Korean words and the semantic fluency task was replaced by a phonemic fluency task. One point was added for subjects with 6 years or less of education in MoCA-K to account for the large illiteracy in elderly Koreans [12]. In the current study, however, we did not add the correction point, to investigate normative data of MoCA-K without any adjustment.

#### Mini-mental state examination-dementia screening (MMSE-DS)

MMSE is the most commonly used dementia screening tool that can be performed in the relatively short time of 5 to 10 min. The MMSE consists of 30 questions with a maximum score of 30. Higher scores indicate better cognition. The MMSE tests the following 7 cognitive domains: orientation in time and place, memory registration and recall, attention and calculation, and language. In Korea, there are several standardized forms of the MMSE, such as MMSE-K [13], K-MMSE [14], MMSE-KC [15]. However, the present study was conducted using MMSE-DS [16]. MMSE-DS has been developed to reflect the specific characteristics of the Korean elderly population and add cultural sensitivity. Normative data and test accuracy were validated for the Korean elderly population using age, sex, and years of education. Scores under 25 indicate cognitive impairment [16].

#### Korean version of cognitive reserve index questionnaire (K-CRIq)

The CRIq has been developed by Nucci et al. It consists of 20 questions collecting demographic information, the number of years of education, and occupational and leisure activities throughout adulthood [8]. Regarding the years of education (CRI-Education), both formal and non-formal education and training years were included. The working activity (CRI-WorkingActivity) value is divided into 5 levels depending on the cognitive load involved. The leisure activity area (CRI-LeisureTime) is measured by evaluating cognitive activity, except for education and occupation activity, using 17 questions to evaluate the type and frequency of cognitive activity. Considering the effect of aging, scores for each category are obtained using age as an independent variable. The scores of 3 domains are calculated again to an average of 100 and a standard deviation of 15 to obtain the total CRIq score (CRI). Choi et al. reported a Korean version of CRIq normalized to age and sex [17].

### Statistical analyses

Descriptive statistics were used to obtain the mean score and standard deviation for demographic characteristics and scores for MMSE, MoCA, and CRIq. Independent *t*-tests were used to compare the scores of different groups divided by sex. To measure correlations between the demographic variables and MoCA scores, Pearson correlation, and multivariate linear regression analyses (independent variable: age, sex, and years of education) were used. The interactions between age and years of education in MoCA were analyzed by multivariate regression analysis using interaction terms.

Univariate and multivariate linear regression analyses, using a stepwise method, were used to evaluate the correlation between CR and the MoCA and MMSE scores. To avoid multicollinearity, the total CRI score was analyzed separately from subdomains of CRIq. In order to compare the degree to which the MoCA and MMSE scores reflect CR, correlation coefficients were compared using Fisher’s *r*-to-*z* transformation, and regression coefficients were compared using *z* transformation. All statistical analyses were performed using Statistical Package for the Social Sciences (SPSS) version 23.0 (SPSS, Inc., Chicago IL) and statistical significance was defined as *P* <  0.05 (2-tailed).

## Results

### Demographics and clinical characteristics

Table 1 shows the demographic and clinical results of the subjects that participated in this study. CRIq is presented as the total score (CRI) and the 3 subdomains, i.e., CRI-Education, CRI-WorkingActivity, and CRI-LeisureTime. When the results of the male and female subjects were compared, male subjects showed significantly higher scores of CRI, CRI-Education, and CRI-WorkingActivity (*P* <  0.001) than female subjects; female subjects exhibited a significantly higher CRI-LeisureTime score than male subjects (*P* <  0.001).

**Table 1:** Demographic and clinical characteristics

	Male (*n* = 95)	Female (*n* = 126)	Total (*n* = 221)	*P* value
Age (year)	74.60 ± 5.54	73.39 ± 5.79	73.91 ± 5.70	0.118
60–74	50 (52.6%)	74 (58.7%)	124 (56.1%)	
75–90	45 (47.4%)	52 (41.3%)	97 (43.9%)	
Education (year)	10.65 ± 4.61	8.83 ± 4.81	9.61 ± 4.80	0.005
0–6	29 (30.5%)	56 (44.4%)	85 (38.5%)	
7–12	35 (36.8%)	38 (30.2%)	73 (33.0%)	
13-	31 (32.6%)	32 (25.4%)	63 (28.5%)	
MMSE	27.47 ± 1.83	26.94 ± 2.23	27.17 ± 2.08	0.041
MoCA	23.40 ± 3.21	22.03 ± 3.65	22.62 ± 3.53	0.004
CRIq				
CRI	107.32 ± 18.46	99.28 ± 16.10	102.74 ± 17.57	0.001
CRI-Education	106.11 ± 15.63	99.30 ± 16.04	102.23 ± 16.19	0.002
CRI-WorkingActivity	114.50 ± 20.70	92.32 ± 11.55	101.86 ± 19.49	< 0.001
CRI-LeisureTime	95.44 ± 16.32	106.80 ± 19.13	101.92 ± 18.80	< 0.001

Data are shown in mean ± standard deviation or number (%)

*MMSE* Mini-mental Status Examination, *MoCA* Montreal Cognitive Assessment, *CRIq* Cognitive Reserve Index questionnaire

### Relationships between demographical variables and MoCA

Table 2 shows descriptive data of MoCA scores using age, sex, and educational level. MoCA scores were associated with all 3 demographic variables, showing higher scores for subjects with longer educational times (correlation analysis: *r* = 0.446, *P* <  0.001), younger age (correlation analysis: *r* = − 0.347, *P* <  0.001), and male subjects (independent *t*-test: *t* = 2.903, *P* = 0.004).

**Table 2:** Mean, standard deviation, and selected percentiles of the MoCA-K by age, educational level, and sex in the normal Korean elderly

Educational level (year)	Male (*n* = 95)			Female (*n* = 126)		
	0–6	7–12	13-	0–6	7–12	13-
Age (year)						
60~ 74						
N	16	19	15	33	24	17
Mean	23.44	24.05	25.33	21.88	23.54	25.12
Standard deviation	2.80	3.47	1.92	3.55	2.89	2.37
Lower quartile	21.25	22.0	24.0	19.0	21.0	23.0
Median	23.0	25.0	26.0	23.0	23.50	26.0
Upper quartile	25.50	27.0	27.0	24.50	26.0	26.0
75~ 90						
N	13	16	16	23	14	15
Mean	19.69	22.50	24.69	18.87	19.71	23.47
Standard deviation	2.29	3.25	2.27	3.08	2.76	2.95
Lower quartile	18.50	19.25	23.0	15.0	18.0	21.0
Median	19.0	23.0	25.0	20.0	19.0	24.0
Upper quartile	21.0	24.75	26.0	21.0	22.0	26.0

*MoCA-K* Montreal Cognitive Assessment

When the demographic variables were analyzed using multivariate linear regression analysis, education level showed moderating effect on the influence of age on MoCA score. The higher the education level was, the lower the degree of MoCA score decreased with age (*B* = 0.017, *P* = 0.023, Additional file 1: Table A1).

### Correlations of MoCA, MMSE, and CR

Pearson correlation was performed between MoCA, MMSE and CRIq scores (Table 3).

**Table 3:** Correlation analyses between CRIq score and MMSE or MoCA scores

	CRI	CRI-Education	CRI-WorkingActivity	CRI-LeisureTime
MoCA	*r* = 0.383 *P* < 0.001	*r* = 0.356 *P* < 0.001	*r* = 0.246 *P* < 0.001	*r* = 0.224 *P* = 0.001
MMSE	*r* = 0.373 *P* < 0.001	*r* = 0.379 *P* < 0.001	*r* = 0.165 *P* = 0.014	*r* = 0.268 *P* < 0.001

*CRIq* Cognitive Reserve Index questionnaire, *MMSE* Mini-mental State Examination, *MoCA* Montreal Cognitive Assessment

Correlation of MoCA score with total CRI was *r* = 0.383, *P* <  0.001; with CRI-Education *r* = 0.356, *P* <  0.001; CRI-WorkingActivity *r* = 0.246, *P* <  0.001, and CRI-LeisureTime *r* = 0.224, *P =* 0.001). Semi-partial correlation analysis adjusting sex was performed in order to find relation between MoCA, MMSE, and CRIq scores adjusting the effect of sex because age and years of education were already adjusted in CRIq and it showed comparable results (Additional file 1: Table A2).

Linear regression analyses were performed to investigate the effect of CRIq on the MoCA and MMSE scores (Table 4).

**Table 4:** Univariate and multivariate regression analyses between CRIq score and MoCA and MMSE scores

Dependent variable	Independent variable	*B*	Standard Error	*t*	*P* value
Univariate regression analyses					
MoCA	CRI	0.076	0.012	6.14	< 0.001
	CRI-Education	0.077	0.014	5.64	< 0.001
	CRI-WorkingActivity	0.044	0.012	3.76	< 0.001
	CRI-LeisureTime	0.042	0.012	3.39	0.001
MMSE	CRI	0.044	0.007	5.95	< 0.001
	CRI-Education	0.049	0.008	6.05	< 0.001
	CRI-WorkingActivity	0.018	0.007	0.01	0.014
	CRI-LeisureTime	0.030	0.007	4.11	< 0.001
Multivariate regression analyses^a^					
MoCA	CRI-Education	0.066	0.014	4.59	< 0.001
	CRI-WorkingActivity	0.025	0.012	2.05	0.042
MMSE	CRI-Education	0.049	0.008	6.05	< 0.001

^a^Multivariate linear regression: independent variables are CRI-Education, CRI-WorkingActivity, and CRI-LeisureTime. The variables included in the final models of multivariate regression analyses using stepwise method are presented

*CRIq* Cognitive Reserve Index questionnaire, *MoCA* Montreal Cognitive Assessment, *MMSE* Mini-mental State Examination

We conducted univariate linear regression analyses for total and each subdomain score of CRIq, and multivariate linear regression analyses using a stepwise method based on the 3 subdomains of CRIq. Total CRI and the 3 subdomains of CRIq were analyzed separately to avoid multicollinearity. The results showed a significant association of the total CRI and MoCA scores (*B* = 0.076, *P* <  0.001), with an explanatory power of 15% (*R*^*2*^ = 0.147, *F* = 37.723, *P* <  0.001). In the final model of multivariate regression analysis using stepwise method (*F* = 18.245, *P* <  0.001), CRI-Education and CRI-WorkingActivity also showed correlations with the MoCA score (CRI-Education: *B* = 0.066, *P* <  0.001; CRI-WorkingActivity: *B* = 0.025, *P* = 0.042), with an explanatory power of 14% (*R*^*2*^ = 0.143). CRI-LeisureTime was not included in the final regression model. For the MMSE score, univariate linear regression analysis (*F* = 35.416, *P* < 0.001) indicated that the total CRI was significantly correlated with the MMSE score (*B* = 0.044, *P* = 0.042) and the explanatory power was 14% (*R*^*2*^ = 0.139). When all 3 domains of CRIq were treated as independent variables in the multivariate linear regression analysis (*F* = 36.655, *P* < 0.001), only CRI-Education was included in the final model with significant effects (*B* = 0.049, *P* < 0.001). The explanatory power was 14% (*R*^*2*^ = 0.143).

### Comparison between MoCA and MMSE on reflection of CRIq

**Fig. 1:**
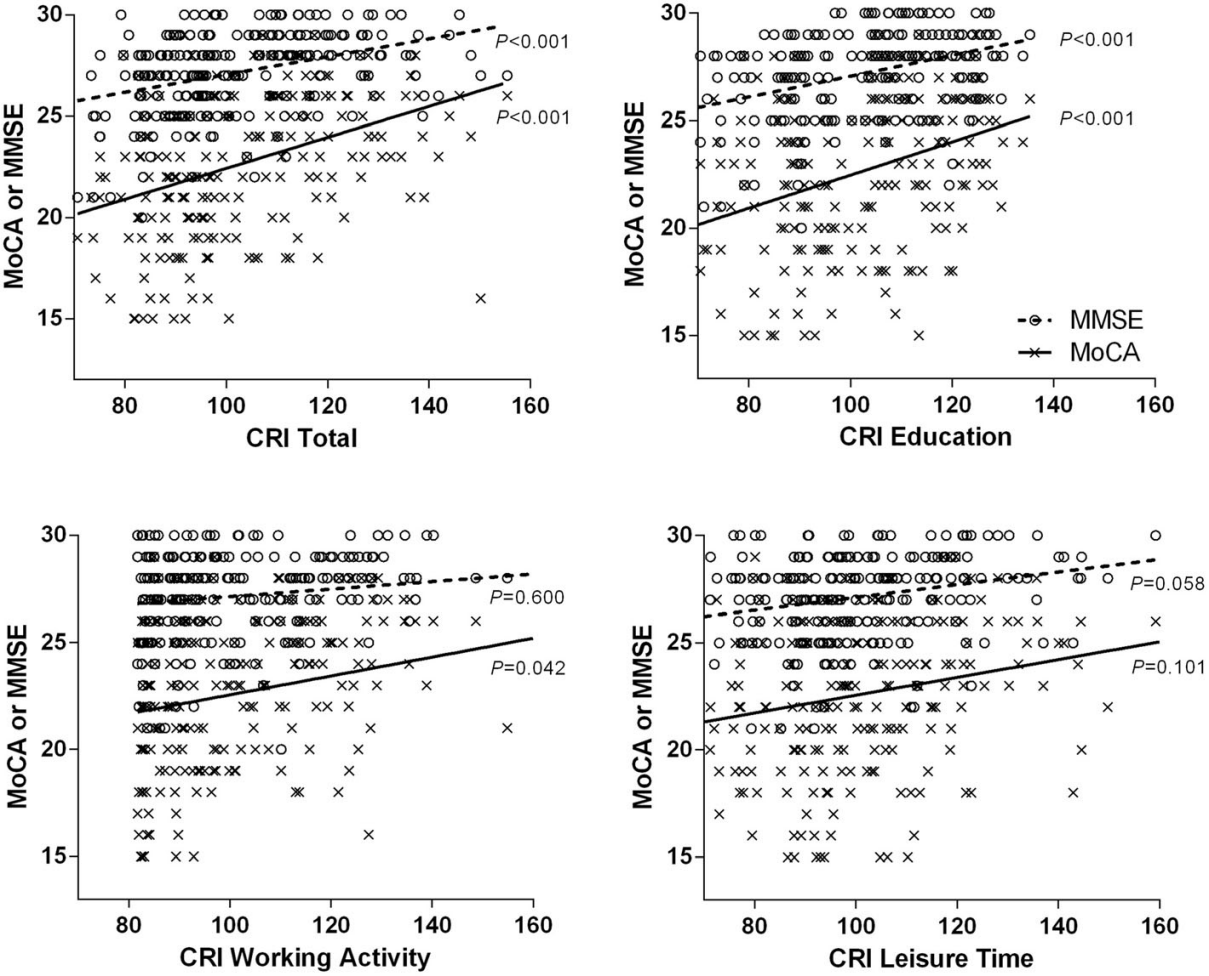
Correlation between MoCA or MMSE and CRIq. *P* values for the subdomains of CRIq are obtained from the multivariate regression analyses using stepwise method (dependent variable: MoCA or MMSE, independent variables: CRI-Education, CRI-WorkingActivity, CRI-LeisureTime). *MoCA* Montreal Cognitive Assessment, *MMSE* Mini-Mental State Examination, *CRIq* Cognitive reserve index questionnaire

Correlations of MoCA and MMSE scores with CRIq are shown in a scatter plot (Fig. 1). The zero-order correlation coefficients shown in Table 3 were compared using Fisher’s *r*-to-*z* transformation. We observed no significant difference in total CRI (*r* for MoCA = 0.383, *r* for MMSE = 0.373) and CRI-Education (*r* for MoCA = 0.356, *r* for MMSE = 0.379). In addition, we compared the multivariate regression coefficient *B* shown in Table 4, which represents the correlation slope between the CRIq score and MoCA or MMSE scores; MoCA scores tended to show a larger slope than MMSE scores. We observed a significant difference in total CRI (*B* for MoCA = 0.076, *B* for MMSE = 0.044, *Z* = 2.30) but not in CRI-Education (*B* for MoCA = 0.066, *B* for MMSE = 0.049, *Z* = 1.06).

## Discussion

In this study, the MoCA was associated with years of education, age, and sex. In addition, MoCA was also significantly associated with CR. Furthermore, we compared the degree to which CR was reflected in the MoCA and MMSE. Our results showed that the MoCA score reflected CR better than the MMSE score.

The first findings of our study are the MoCA scores. MoCA scores tended to be higher for the patients with more years of education and of younger age. These results are similar to results from previous normative studies [18, 19]. Additionally, aging had a larger effect on MoCA scores in a population with lower education than in a population with higher education. This result indicates that the effect of education overcomes the effect of aging. This is in line with previous studies suggesting that education is a major factor in CR [20, 21]. Regarding this significant effect of education, Nasreddine et al. have included one correction point in the MoCA for individuals with education of 12 years and below. In Korea, Lee et al. set a similar correction point in the MoCA-K for individuals with 6 years or less of education, considering the low level of education in Korea [12]. However, in this study, the correction point was not applied to determine the association between normative scores of MoCA-K and CR or demographic variables. MoCA scores were higher in male subjects than in female subjects, which is in line with the results of the Chinese MoCA study [22] and an MMSE normative study performed on Koreans [23]. This sexual discrepancy in normative data is considered to reflect a tendency of elderly men to have more intellectual, social, and physical opportunities than women due to gender role differences.

Our results demonstrated that both MoCA and MMSE correlate with CR, although MoCA score reflects CR more sensitively than MMSE score. In regression analyses with total and subdomain scores of CRIq, the MoCA score reflected total CRI, CRI-Education, and CRI-WorkingActivity, while the MMSE score only reflected total CRI and CRI-Education. In addition, the correlation slope between the total CRI and MoCA scores was significantly higher than that between the total CRI and MMSE scores. We suggest that this discrepancy was due to differences in the tools of assessment employed by the MoCA and MMSE; MoCA contains various assessment tools for frontal lobe function (TMT-B, copy of a cube, CDT, letter A tap, letter fluency), which are not included in MMSE, making it sensitive to and reflective of CR in various cognitive subdomains [24]. It is known from previous studies that connectivity in the frontal lobe plays an important role in CR [25, 26]. This can be more prominent in the elderly. Compared to young individuals, old individuals use different brain networks [27]. Scarmeas et al. investigated the brain regions related to CR and found that the inferior frontal region is related to CR only in old subjects [28]. The increase in activity and connectivity in the prefrontal area of patients with AD, compared with normal controls, has been interpreted to reflect the recruitment of cognitive resources [9, 29]. Increased activity in the prefrontal cortex has also been associated with tasks such as episodic, retrieval, and recognition memory, which are the most basic memory functions and are frequently affected by cognitive decline [25].

In particular, our study suggests that only the MoCA scores can sensitively reflect CRI-WorkingActivity among the subdomains of CRIq. This association between MoCA and the vocational ability is attributed to the assessment of frontal lobe function by MoCA. The effect of vocational ability on CR can be explained by the motivation to participate in cognitively stimulating daily activities, neuronal plasticity, and executive functions, making it a favorable domain to examine CR [30, 31]. Many studies have shown that vocational abilities are dependent on frontal lobe function in patients with traumatic brain injuries and vascular degenerative changes. Since the frontal lobe is involved in language, arithmetic processing, attention, planning and strategy application, and willful action, it can be a good indicator of vocational ability [32–34]. Proxies such as educational or occupational achievements, and IQ have been used to characterize CR previously [7, 31]. In conjunction with our results, it appears that it would be clinically beneficial to use MoCA as a brief cognitive screening tool for the assessment of both cognitive function and reserve.

Recently, interest in CR has increased because of its importance in identifying and managing patients with preclinical and prodromal AD. Lacking a current disease-modifying treatment, CR can be another interesting candidate for the prevention and treatment of AD. Accordingly, the number of studies investigating treatments using cognitive stimulation is sharply increasing [35]. CR consists of these lifetime experiences. High levels of CR have been reported to be capable of lowering the risk of incidents of dementia, its clinical symptoms, and its pathologic changes, as shown by neuroimaging studies [6, 10, 36–38].

Our study shows that MoCA score correlates with CR, especially in terms of education and working activity, which corresponds to executive function. Therefore, MoCA can be a useful tool to evaluate CR and to screen the subtle changes in cognition. However, our present study has limitations that should be taken into account when evaluating the results. For example, our study only included a small number of participants, and the participants in this study had fewer years of education than those in studies from other countries. Future larger studies supported by more validating methods and biological assays are required to overcome these limitations.

## Conclusion

This study confirms that MoCA reflects CR, and that CR is reflected more sensitively by the MoCA score than the MMSE score. The clinical use of the MoCA is expected to increase markedly, because it provides an easy way to evaluate cognitive function and CR, without any additional tests or large-scale batteries. This study may provide valuable insight for future, large community-based studies of early cognitive decline and CR.

## Additional file

Additional file 1: **Table A1.** Moderating effect of Education on the relationship between Age and MoCA score. **Table A2.** Semi-partial correlation between CRIq and MMSE or MoCA scores adjusting sex. (DOCX 19 kb)

## Abbreviations

CDT: Clock-Drawing Test
CR: Cognitive Reserve
CRIq: Cognitive Reserve Index questionnaire
DSM-IV: Diagnostic and Statistical Manual of Mental Disorders, fourth Edition
IQ: Intellectual Quotient
MCI: Mild Cognitive Impairment
MMSE: Mini-Mental State Examination
MoCA: Montreal Cognitive Assessment
TMT-B: Trail-Making Test, Part B
