# Supplementary material for: Montreal cognitive assessment reflects cognitive reserve
Source: BMC Geriatr. 2018 Oct 30;18:261. doi: 10.1186/s12877-018-0951-8 (PMC6208087; doi:10.1186/s12877-018-0951-8)
Supplement: Supplementary file 1 — Table A1. Moderating effect of Education on the relationship between Age and MoCA score. Table A2. Semi-partial correlation between CRIq and MMSE or MoCA scores adjusting sex. (DOCX 19 kb) [file 12877_2018_951_MOESM1_ESM.docx]

| **Table A1**. Moderating effect of Education on the relationship between Age and MoCA score. | | | | | | | | | |
| --- | --- | --- | --- | --- | --- | --- | --- | --- | --- |
|  | **1^st^ model** | | | |  | **2^nd^ model** | | | |
|  | **B** | **SE** | ***β*** | ***P*** |  | **B** | **SE** | ***β*** | ***P*** |
| Age (years) | -0.214 | 0.034 | -0.349 | < 0.001 |  | -0.398 | 0.087 | -0.648 | <0.001 |
| Sex (male 1, female 2) | -1.060 | 0.401 | -0.150 | < 0.001 |  | -1.042 | 0.397 | -0.148 | 0.009 |
| Education (years) | 0.296 | 0.041 | 0.406 | 0.009 |  | -0.990 | 0.565 | -1.359 | 0.081 |
| Age*Education |  |  |  |  |  | 0.017 | 0.008 | 1.784 | 0.023 |
| Model fitness | *F* = 35.745, *P* < 0.001  *R^2^* = 0.331 | | | |  | *F* = 28.631, *P* < 0.001  *R^2^* = 0.346 | | | |
| Multivariate linear regression analysis (independent variables as age, sex, and years of education, dependent variable: MoCA score)  MoCA: Montreal Cognitive Assessment, SE: standard error | | | | | | | | | |

| **Table A2**. Semi-partial correlation between CRIq and MMSE or MoCA scores adjusting sex | | | | |  |
| --- | --- | --- | --- | --- | --- |
|  | **CRI** | **CRI-WorkingActivity** | **CRI-**  **Education** | **CRI-LeisureTime** | |
| MoCA | *r* = 0.356  *P* < 0.001 | *r* = 0.172  *P* = 0.011 | *r* = 0.330  *P* < 0.001 | *r* = 0.299  *P* < 0.001 | |
| MMSE | *r* = 0.356  *P* < 0.001 | *r* = 0.114  *P* = 0.092 | *r* = 0.363  *P* < 0.001 | *r* = 0.323  *P* < 0.001 | |
| CRIq: Cognitive Reserve Index questionnaire, MMSE: Mini-mental State Examination, MoCA: Montreal Cognitive Assessment | | | | |  |
